# Do regional tax incentive policies improve productivity?

**DOI:** 10.1371/journal.pone.0307561

**Published:** 2024-08-27

**Authors:** Mengchao Zhao, Xiang Xiao, Jiaan Yang, Haonan Chen

**Affiliations:** 1 School of Economics, Capital University of Economics and Business, Beijing, China; 2 School of Marxism, Central University of Finance and Economics, Beijing, China; Zhanjiang University of Science and Technology, CHINA

## Abstract

The difficulty in transforming old industrial areas constitutes a significant factor contributing to regional development imbalances. Can regional tax incentives, as a crucial component of regional policies, polish the “rust belt” regions? This study leverages the inaugural Value-Added Tax (VAT) reform in China as an opportunity to explore the potential of regional tax incentives in achieving sustainable development in traditional industrial areas. Drawing upon a comprehensive industrial enterprise database, we employ a Propensity Score Matching-Difference in Differences (PSM-DID) approach to examine the efficacy of these tax incentives. Our findings reveal that: (1) Regional tax incentives primarily enhance firms productivity by stimulating investment in enterprises, yet they do not contribute to improved investment efficiency or spur innovation within firms. (2) Regional tax incentives have alleviated financing constraints for enterprises in old industrial bases, significantly enhancing the Total Factor Productivity (TFP) of firms with higher financing constraints. This policy has had an even stronger impact on improving the TFP of state-owned and monopolistic enterprises. (3) Regional tax incentives have impeded productivity growth by preventing the exit of low-efficiency firms and the entry of high-efficiency ones. These incentives also increased the likelihood of “zombie firms” forming and failed to promote endogenous economic growth in the Northeast region. Additionally, they have distorted the allocation of resources towards capital and technology-intensive industries in that area. In China’s old industrial bases, regional tax incentives should be coordinated with market-oriented reforms; these regional tax incentive policies should also be further enriched.

## Introduction and literature review

Global experiences show that traditional industrial areas like the US Great Lakes Industrial Region, which were once leaders in industrialization, often face manufacturing declines and are known as “rust belts” [1]. In the early 1990s, China’s Northeast, an old industrial base, exhibited signs of sluggish economic growth (EndNote 1 in S1 Data). In response, in 2003, China launched the “Revitalize the Northeast” strategy, with VAT reform as a key policy tool. This strategy led to a period during 2004–2013 where the economic growth rate of the Northeast exceeded the national average, though a precipitous decline occurred in 2014 (EndNote 2 in S1 Data). Studying whether regional tax incentives can achieve sustainable development in the Northeast region not only enriches theoretical understanding of the economic performance of regional tax incentives but also provides Chinese experience in revitalizing the “Rust Belt”.

Regional tax incentives are a crucial component of regional policy. Scholars, primarily focusing on the conditions of Western developed countries, argue that regional policies can foster economic agglomeration in less developed areas, enhancing employment levels [2, 3], and improving local TFP [4]. These policies are also beneficial for economic development [5, 6]. However, the economic performance of regional policies in developing countries may vary significantly [7]. Some recent have conducted in-depth discussions on regional policies in developing countries. Abeberese and Chaurey(2019) [8] argue that while regional policies may benefit the growth of formal enterprises in India, they adversely affect the increase in informal enterprises, ultimately leading to a decline in the total number of enterprises. Olfert et al. (2014) [9] noted that regional tax incentives effectively stimulated economic development in India’s less developed areas, while Hasan et al. (2021) [10] highlighted the positive impact on new enterprise entry in India’s disadvantaged regions. Halleck Vega (2021) [11] pointed out that although regional policies promoted industrialization in Brazil’s economically lagging areas, their lasting effects were limited.

In regional economics, the study of “rust belts” remains a weak link [12]. Compared to underdeveloped areas, old industrial bases boast strong industrial foundations, comprehensive infrastructure, and substantial human capital. However, their disadvantage lies in a strong “path dependency” on traditional industrial models. Characterized by heavy industries, “Rust Belts” face issues like a singular industrial structure, lack of technological advancement, rising costs, severe population loss, and deteriorating infrastructure [13–15]. China’s Northeast, a typical old industrial base, shares challenges similar to the developed world’s “Rust Belts” but also has unique features like a high concentration of large state-owned enterprises, underdeveloped markets, and a focus on basic heavy industries. Revitalizing old industrial bases often requires government intervention [16]. Thus, the impact of regional tax incentives, a key governmental policy tool, on China’s Northeastern old industrial base warrants further discussion in light of China’s specific conditions.

Although many scholars believe that the VAT reform in the Northeast region has promoted local economic development and facilitated the transformation of economic development modes [17–20], some argue that the impact of VAT reform on the development of the Northeast region is limited. Key issues such as underdeveloped markets, lack of regulations, and difficulties in industrial transformation are significant factors hindering the economic revival of the Northeast [21–24]. The study by Yu and Qi (2022), which closely relates to our paper, found that VAT reform helps improve enterprise total factor productivity [25]. However, it failed to objectively assess the performance of tax incentive policies in a comprehensive analysis of multiple pathways. Research on whether regional tax incentives can improve the intrinsic resource endowments of enterprises, thereby generating endogenous growth drivers for regional economic growth, is still lacking. Furthermore, few studies have connected the productivity Dynamic Olley-Pakes Decomposition (DOP decomposition) proposed by Melitz and Polanec (2015) [26] with tax incentive policies to thoroughly quantify the impact of tax incentives on regional resource allocation efficiency. In general, existing research has tended to overlook the long-term sustainability perspective when considering the implementation effects of tax incentive policies. This is precisely the issue that this paper aims to address.

This paper systematically discusses the productivity effects of regional tax incentives. Through empirical research, it suggests that regional tax incentives may exacerbate “path dependency” in old industrial bases, intensifying the challenges associated with both institutional and industrial transformations in these areas. While tax incentives may enhance firms’ capital-embedded productivity and temporarily mask existing contradictions, they also result in inefficient investment, insufficient innovation, and distorted resource allocation. These problems lay the groundwork for a steep decline in the future. The main contributions of this paper are: (1) an in-depth discussion of the impact and mechanisms of regional tax incentives on the productivity of old industrial bases as a specific area, identifying the primary channels through which tax incentives enhance productivity; (2) the incorporation of the DOP decomposition into the framework for assessing the economic performance of regional tax incentives, discussing their impact on the aggregate productivity of manufacturing and its components from the perspective of enterprise dynamics; (3) an attempt to provide a theoretical explanation for the cliff-like decline of the economy in the Northeast’s old industrial bases from a productivity perspective, offering an empirical foundation for promotion of sustainable regional development.

## Institutional background and research hypotheses

### Institutional background

At the dawn of its foundation, influenced by factors such as resource endowment, industrial foundation, and national security, the Northeast region of China became a focal point of investment, heralded as the eldest son of the Republic’s industrialization. The “First Five-Year Plan” and “Second Five-Year Plan” saw a slew of heavy industrial projects established in the Northeast. The industrialization of the Northeast was propelled under government leadership, with large and medium-sized state-owned enterprises as its micro-foundation. Massive fixed asset investments under government guidance effectively drove technological progress in industry. During the “First Five-Year Plan” period, the Northeast region accounted for 50 out of the 106 major civilian projects aided by the Soviet Union. While the Soviet assistance significantly facilitated the industrialization of the Northeast’s old industrial base, it also left a strong soviet imprint on the region.

The government’s vigorous industrialization efforts led to the formation of two pillar industries in the Northeast: the resource industry and the machinery industry. However, post-economic reform and opening up, the Northeast’s industrial development faced severe challenges. On one hand, technological advancements in old industrial base enterprises were largely embedded in massive government investments. With the shift of national investment priorities, many enterprises’ equipment remained outdated for long periods, leading to a gradual decline in industrial efficiency in the region. On the other hand, during the planned economy era, most state-owned enterprises established in the Northeast region primarily served the national priority of developing heavy industry. Their establishment and growth were more aligned with national strategic tasks rather than being based on their inherent resources, resulting in many enterprises lacking viability in market competition after the reform and opening up period [27]. Continuous resource depletion and a heavy policy burden further exacerbated the difficulties faced by enterprises in the Northeast, leading to widespread operational difficulties, slow technological upgrades, and severe talent outflows since the 1990s, further worsening the vicious cycle hindering the region’s development.

**Fig 1:**
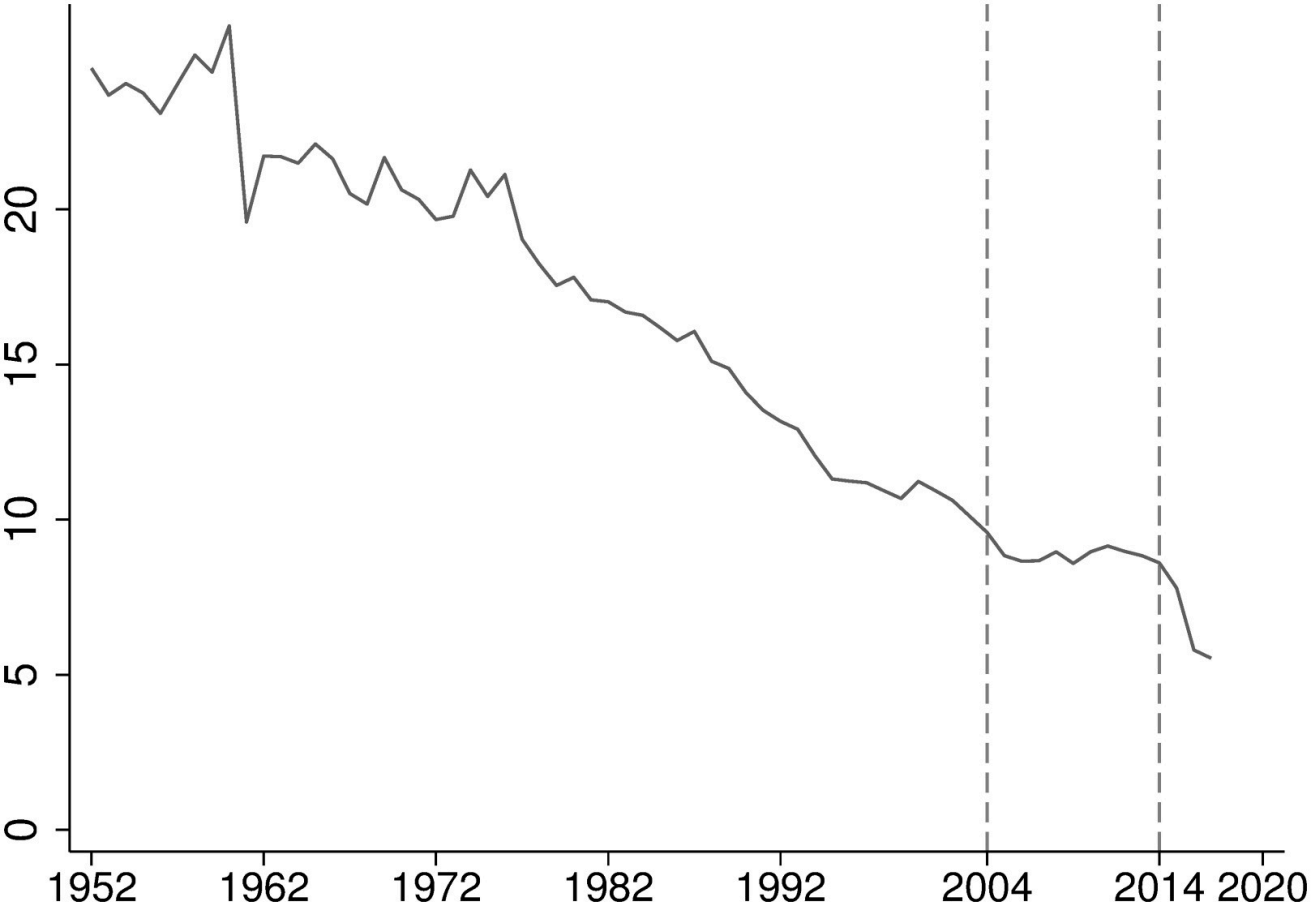
Proportion of industrial output value of the Northeast region in the national total.

In 2003, China introduced the “Revitalize the Northeast” strategy, and various regional preferential policies began to take effect from 2004, with VAT reform in the Northeast emerging as an important component of these tax incentives. Prior to 2004, China employed a production-based VAT system nationwide, allowing enterprises to deduct VAT on purchased raw materials but not on fixed assets acquired externally. This system was particularly disadvantageous for heavy chemical industries, the dominant industry in the Northeast, as it led to double taxation and high costs for technological upgrades. Starting July 1, 2004, China piloted VAT reform in the Northeast, targeting industries such as equipment manufacturing and petrochemicals, allowing enterprises to deduct VAT paid on purchased fixed assets from their taxable amount. This reform alleviated double taxation, reduced enterprises’ burdens, especially lowering the cost of machinery acquisition, and encouraged technological upgrades. With a series of government measures, including tax incentives, the sluggish development of the manufacturing industry in the Northeast region has been somewhat alleviated. However, a severe industrial downturn occurred post-2014, leading to an economic cliff-like fall (Fig 1). A comprehensive assessment of regional tax incentives within the “Revitalize the Northeast” strategy not only offers lessons for revitalizing old industrial bases in emerging nations but also provides empirical evidence for the applicability and scope of regional tax incentives.

### Theoretical analysis and research hypotheses

Regional tax incentives are a crucial governmental tool for intervening in the economic development of underdeveloped areas and a hot topic in economic research. Such incentives might enhance the cash flow of existing businesses in supported areas, easing financial constraints. Technological advancement embedded within capital deepening is a significant characteristic of China’s economic growth. For old industrial bases, investing in machinery and equipment to drive technological progress and enhance productivity is an essential development model [28]. In the Northeast, where financial markets are less developed, VAT deductions not only reduce corporate burdens and stimulate investment vitality but also encourage the purchase of machinery and equipment, promoting fixed asset investment and technological upgrades, thereby enhancing TFP.

Compared to direct fiscal investments by the government in old industrial bases, regional tax incentives have more market-oriented characteristics and are inclusive, leaving more decision-making power to businesses. They are a commonly used tool in regional policies. However, due to the old industrial bases’ strong “path dependency” on previous development models [29], the productivity-enhancing effect of regional tax incentives may be limited. Given the characteristics of the Northeast, the adverse effects of regional tax incentives on corporate productivity may initially be masked by capital investments that incorporate technological advancements, thus appearing to boost productivity. Therefore, we propose the hypothesis 1.

H1: Tax incentive policies increase the total factor productivity of enterprises.

Regional tax incentives promote corporate investment. The old industrial base in Northeast China, which emerged during the planned economy period, has been a stronghold of industrialization in new China, with heavy chemical industry as its pillar and large-to-medium state-owned enterprises as its micro-foundation, relying heavily on national-level large-scale investments for economic development. The practice of making large-scale purchases of advanced machinery and equipment for leapfrogging technological advancements has been a significant feature of Northeastern industrial development [30]. However, given the strong planned economy characteristics of the Northeast region and the significant influence of the corporate culture of traditional state-owned enterprises, many businesses still suffer from the chronic issue of “soft budget constraints” [31]. While regional tax incentives reduce the costs of business expansion, they may also further intensify their “investment hunger” [31], and excessive inefficient investment could lower investment efficiency.

Technological progress is key to the long-term productivity growth of old industrial bases. Many studies suggest that tax incentives alleviate corporate cash flow issues, which can foster innovation [32]. However, due to the strong organizational inertia [33] and path dependency [29] of enterprises in the Northeast’s old industrial base, regional tax incentives may more likely promote the acquisition of advanced technological equipment rather than higher-risk technological innovation. Autonomous innovation carries significant uncertainties, and for later-developing regions, purchasing ready-made advanced machinery and equipment offers a higher certainty and controllability of investment returns. Based on the above analysis, we propose the following hypothesis 2.

H2: Tax incentives primarily enhance total factor productivity by promoting investment scale, rather than autonomous innovation within enterprises.

Regional tax incentives may reinforce the planned economic system of the old industrial area in Northeast China. Originating during the planned economy period, the Northeast’s old industrial base has a high proportion of state-owned enterprises, some of which also exhibit monopolistic characteristics. On one hand, these enterprises have intricate connections with local governments, embodying a form of paternalism [34]. As a specific economic organizational form in socialist countries, state-owned enterprises carry multiple objectives, including upholding the political system, achieving government political goals, and fulfilling social construction tasks [35]. Local governments also tend to prioritize supporting these state-owned enterprises. On the other hand, regional tax incentives may further ease the financing constraints for these enterprises, stimulate their expansion, and enhance their relative standing in the Northeast. Therefore, we propose the following hypothesis 3.

H3: Regional tax incentives have a more significant impact on the total factor productivity of state-owned and monopolistic enterprises.

Optimizing resource allocation is an important way to enhance manufacturing efficiency and ensure sustainable development in regions [36–38]. Existing literature suggests that regional tax incentives, by relatively reducing the marginal cost of capital, can attract high-productivity enterprises, create agglomeration economies, facilitate the sharing of relevant information and resources, promote the development of the intermediate goods market, and generate knowledge spillover effects, thus benefiting the regional production efficiency [39–41].

However, considering the specific nature of old industrial bases, regional tax incentives might lead to distortions in resource allocation efficiency. Under market competition, the expansion of high-efficiency enterprises and the elimination of low-efficiency ones should concentrate more resources on high-efficiency enterprises, thereby improving overall economic efficiency [27]. However, tax incentives supporting specific regions and industries might further obscure the contradictions in industrial and institutional transformation in old industrial bases. Many struggling enterprises, incentivized by tax breaks, might increase investments and temporarily enhance productivity through technological upgrades, allowing them to continue operating. Yet, the management inefficiencies of these low-efficiency enterprises are masked by the productivity gains from large-scale investments, which not only hinders the optimization of resource allocation but also exacerbates the difficulties in economic transformation of old industrial bases. Therefore, we propose the hypothesis 4.

H4: Regional tax incentives are detrimental to the optimization of resource allocation and do not facilitate the improvement of manufacturing productivity.

Regional tax incentives may increase the likelihood of creating “zombie firms” in old industrial bases. The marketization reforms in the Northeast old industrial base are still incomplete, and many enterprises continue to suffer from “soft budget constraints” and a strong “investment hunger” [29]. Regional tax incentives, being inclusive, will also ease the financing constraints of these enterprises. Under such tax incentives, many already struggling enterprises may still be motivated to expand their scale. This can increase the risk of these enterprises becoming “zombie firms”, further worsening resource allocation in the old industrial base.

Regional tax incentives might not effectively attract high-efficiency enterprises. Attracting high-efficiency enterprises to enter the target area and form agglomeration economies is a crucial strategy to mitigate the outflow of production factors, including human and capital resources, from old industrial bases [16]. However, the Northeast old industrial base lags behind the eastern coastal regions in terms of marketization and openness, which limits the attractiveness of tax incentives to high-efficiency enterprises. Additionally, since regional tax incentives provide support to low-efficiency enterprises, they might worsen the local business environment, thereby adversely affecting the entry of high-efficiency enterprises. Based on this analysis, we propose the following hypothesis 5.

H5: Regional tax incentives are not conducive to the exit of low-efficiency enterprises nor do they attract the entry of high-efficiency enterprises.

**Fig 2:**
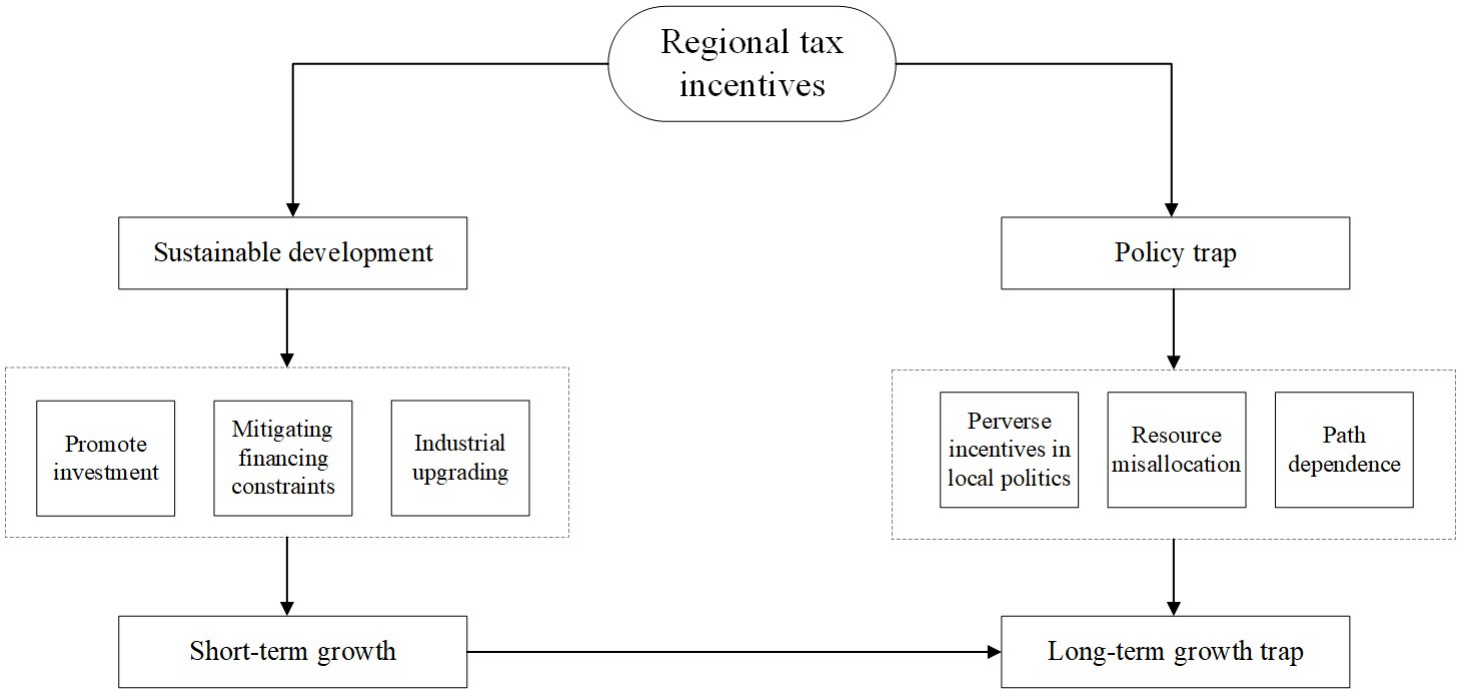
Logic graphs.

To more clearly illustrate the theoretical logic discussed, Fig 2 provides a visual representation.

## Research design and empirical analysis

### Model specification, variable selection, and data source

#### Model specification

This paper treats the 2004 VAT reform targeted at six major industries in the Northeast region as a quasi-natural experiment (EndNote 3 in S1 Data). It employs a DID approach to examine the policy’s impact on firms’ TFP, establishing the following baseline model:

$$\begin{array}{c} TFP_{ijkt}=\alpha_{0}+\alpha_{1}Post+\alpha_{2}Treat+\alpha_{3}Treat\times Post+AX+\theta_{i}+\gamma_{t}+\varepsilon_{ijkt} \end{array}$$ (1)

Given the significant regional development disparities in China, the DID method’s requirement for a consistent time trend assumption between the treatment and control groups is stringent. To address this, the study utilizes PSM to mitigate sample selection bias before implementing DID, resulting in a more accurate evaluation of the VAT reform’s effects through a PSM-DID model:

$$\begin{array}{c} TFP_{ijkt}^{PSM}=\alpha_{0}+\alpha_{1}Post+\alpha_{2}Treat+\alpha_{3}Treat\times Post+AX+\theta_{i}+\gamma_{t}+\varepsilon_{ijkt} \end{array}$$ (2)

In Eqs (1) and (2), the indices *i*, *j*, *k* and *t* represent the firm, industry, region, and year, respectively. TFP and TFP^*PSM*^ denote the TFP of firms before and after PSM, respectively. The variable Post is a dummy indicating the implementation of VAT reform, assigned a value of 1 for samples from 2004 onwards, and 0 otherwise. Treat is a treatment group dummy, assigned a value of 1 for samples located in the Northeast region and within one of the six major industries, otherwise 0. *X* comprises a set of control variables covering both the firm and provincial levels. *θ* signifies firm fixed effects, *γ* represents year fixed effects, and *ε* is the error term that varies over time and individuals. The interaction term Treat×Post, a variable of primary interest, assesses the impact of VAT reform on firms’ TFP. A significantly positive *α*_3_ indicates that VAT reform has contributed to an enhancement in the TFP of manufacturing firms.

In the DID model, the meanings of each parameter can be found in Table 1, for enterprises that implemented the tax incentive policy (trea = 1), the changes in productivity before and after the policy implementation are given by *α*_0_ + *α*_2_ and *α*_0_ + *α*_1_ + *α*_2_ + *α*_3_, respectively. The change in productivity due to the implementation of the tax incentive policy for these enterprises is *α*_1_ + *α*_3_, which includes the impact of the tax incentives as well as other factors. Conversely, for enterprises that did not implement the tax incentive policy (trea = 0), the changes in productivity before and after the policy are *α*_0_ and *α*_0_ + *α*_1_, respectively. The change in productivity for these enterprises, which did not benefit from the tax incentives, is *α*_1_. This difference does not include the impact of the tax incentive policy. Therefore, by subtracting the change in productivity of the control group (*α*_1_) from the change in productivity of the treatment group (*α*_1_ + *α*_3_). We can calculate the net impact of the tax incentive policy on enterprise productivity (*α*_3_).

**Table 1:** Explanation: Impact of tax incentive policies on productivity.

	Before the Implementation of the Tax Incentive Policy (post = 0)	After the implementation of the tax incentive policy (post = 1)	Difference
Test Group (trea = 1)	*α*_0_ + *α*_2_	*α*_0_ + *α*_1_ + *α*_2_ + *α*_3_	*α*_1_ + *α*_3_
Control Group (trea = 0)	*α*_0_	*α*_0_+ *α*_1_	*α*_1_
Difference-in-difference			*α*_3_

A critical prerequisite for employing the DID method is the assumption of common trends between the treatment and control groups. This assumes that, absent the tax incentive policy, there would be no systematic difference in the trend of productivity changes over time between the treated and control group enterprises. The PSM method calculates the probability of each individual receiving the intervention and matches individuals in the treatment group with those in the control group based on these scores. This matching aims to ensure that both groups are similar in observed covariates before the intervention, making the pre-intervention trends more comparable between the two groups. However, PSM only constructs a new control group and does not quantify the policy effect. In contrast, the PSM-DID method, proposed and developed by Heckman et al. (1997) [42], can eliminate sample selection bias and evaluate policy effects, effectively addressing the limitations of both the PSM and DID methods. Additionally, we further validate the robustness of our estimation results through Robustness Checks.

The PSM process unfolds as follows: initially, variables such as firm age, ownership type, tax burden, and wage cost are selected as characteristic explanatory variables for regression to calculate propensity scores for each sample. Subsequent to this, the nearest-neighbor matching method, guided by the propensity scores, is employed to pair samples in the treatment group with those in the control group, thereby generating a matched sample set. The outcomes of the PSM matching are then rigorously evaluated.

**Fig 3:**
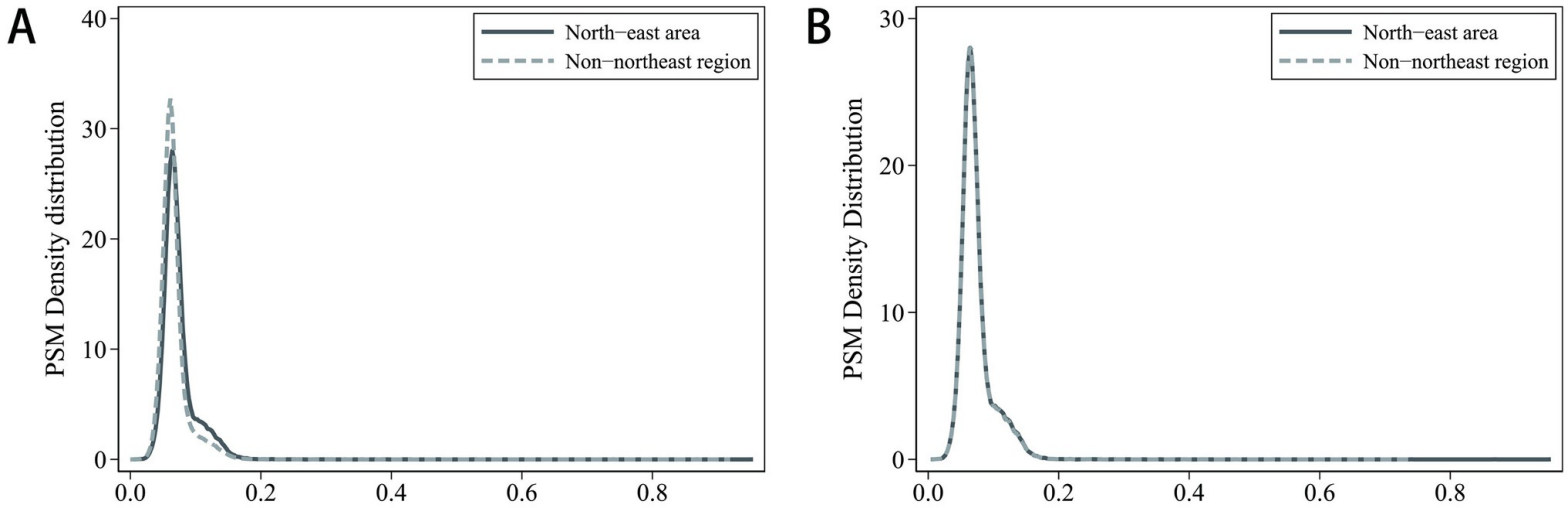
PSM kernel density.

**Fig 4:**
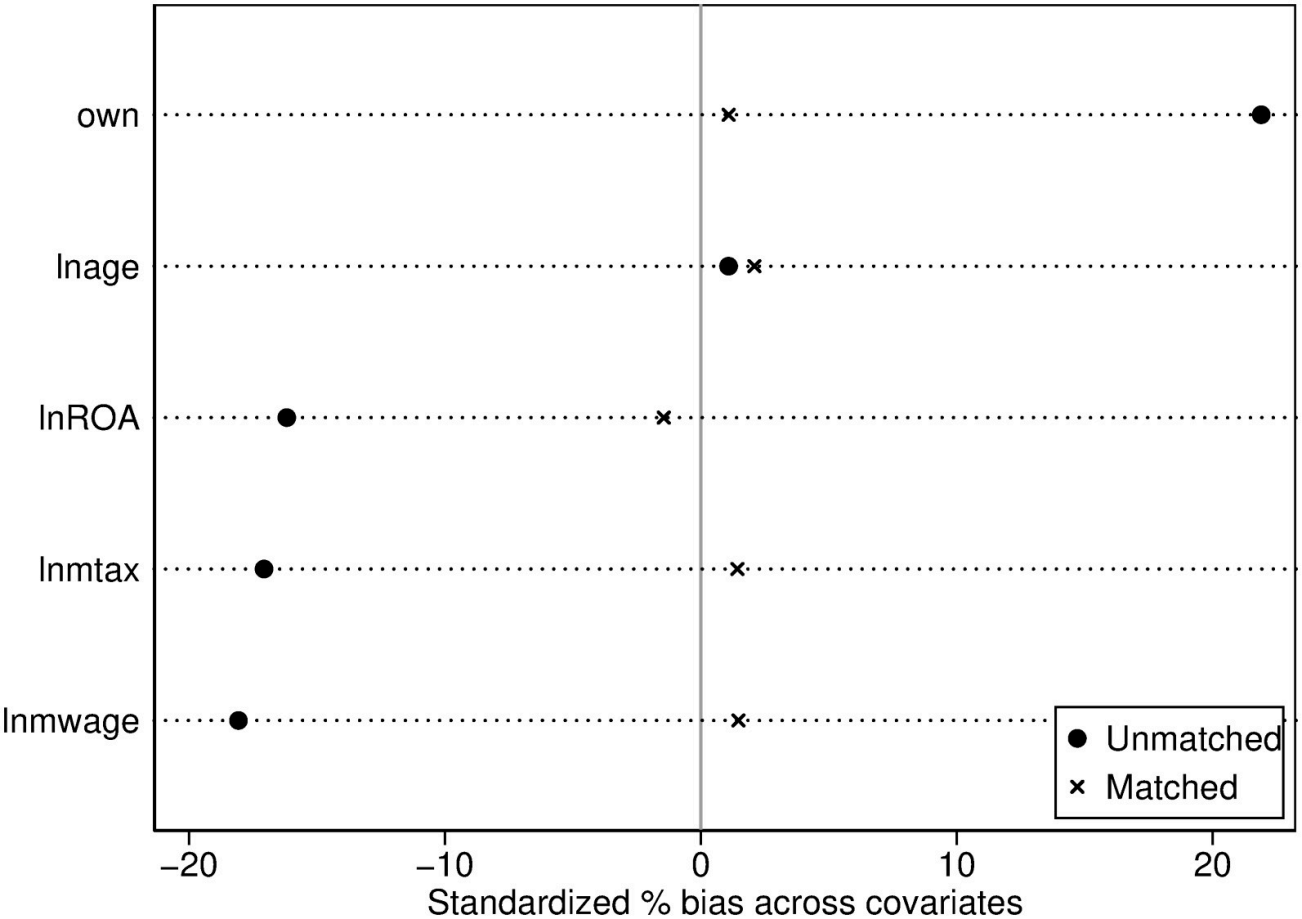
Post matching effect diagram.

Before matching, the propensity score distribution curves between the treatment and control groups exhibited significant differences. However, post-matching, the kernel density curves of the propensity scores for both groups nearly overlap (as seen in Fig 3), and the differences in variables between the matched treatment and control groups are all below 5% (as shown in Fig 4). This indicates a successful matching process, which substantially mitigates endogeneity issues stemming from sample selection, thereby enhancing the reliability of the DID analysis results.

#### Variable selection

- Dependent variable:
  - *TFP*: This study calculates the firm’s TFP using the Levinsohn-Petrin (LP) method.
- Control Variables:
  - Firm-Level Variables:
    - Firm Age (*lnage*): Logarithm of one plus the difference between the current year and the year of establishment, capturing the experience and potential maturity of the firm.
    - State Capital Share (*own*): Ratio of state capital to total paid-in capital, indicating the extent of government ownership.
    - Tax Burden Level (*lnmtax*): Logarithm of one plus the sum of VAT and corporate income tax, reflecting the firm’s tax liability.
    - Wage Cost (*lnmwage*): Logarithm of total wages paid, representing labor cost.
    - Return on Assets (*lnROA*): Logarithm of the ratio of net profit to total assets, measuring profitability and asset efficiency.
  - Province-Level Variables:
    - Economic Development Level (*lngdp*): Logarithm of per capita GDP, indicating regional economic prosperity.
    - Industrialization Level (*TIA*): Proportion of secondary industry output in total output, showing industrial development.
    - Number of Enterprises (*lnN*): Logarithm of total number of firms in each province and industry, reflecting business density.
    - Average Firm Size (*lnscale*): Logarithm of average sales revenue, indicating the scale of operations within industries and provinces.

All economic variables are adjusted for inflation using relevant price indices to ensure accurate comparisons and analyses over time.

#### Data source

Our data primarily comes from the following sources:

- Chinese Firms Data: This data is the annual surveys conducted by the National Bureau of Statistics of China and includes all state-owned industrial enterprises and other industrial enterprises with annual sales exceeding 5 million RMB. It comprises major financial indicators such as assets, sales revenue, profits, number of employees, and wages. The data are collected from quarterly and annual reports submitted by these enterprises to local statistics bureaus. This database is not only widely used in domestic and international economic research but also frequently utilized in policy analysis and formulation [37, 43].
- Statistical Yearbooks: The Statistical Yearbook is an official statistical publication issued by China’s central and regional statistical bureaus. When releasing data, the statistical bureaus typically provide the methodology used for data collection and processing, including the sources of the data, sampling methods, survey techniques, and data processing procedures. This transparency enhances the trustworthiness of the data. Moreover, the use of Statistical Yearbook data in international research and comparative analyses underscores its quality and reliability [44].
- Chinese Patents Data: This data covers all patent applications filed since the enactment of the Patent Law of the People’s Republic of China. The data are sourced from the China National Intellectual Property Administration (CNIPA, formerly SIPO), which is responsible for the examination and registration of patents in China. CNIPA provides detailed information about patent applications on its official website, including the application process, review status, and public patent documents. This availability and transparency help the public and researchers verify and assess the reliability of the data.

Since we focuse on the effects of localized tax reforms, data from a comprehensive rollout are not applicable to this discussion. We use data from 1998 to 2006 to study the impact of regional tax incentive policies on enterprise productivity, employing the VAT transformation reform in the Northeast region as a ‘quasi-natural experiment’. Additionally, since the benefiting enterprises belong to six industries—equipment manufacturing, petrochemical industry, metallurgy, shipbuilding, automobile manufacturing, and agricultural product processing—we extracted eligible sample enterprises based on the “National Economic Industry Classification Codes”. Although the VAT transformation policy was later extended to several hundred military and high-tech enterprises, some of these enterprises partially belong to the aforementioned six industries. Considering the difficulties in identification and sample size, these enterprises were not included in our sample.

Following methodologies established in Brandt et al. (2012) [45], we undertook data cleaning to address issues of missing data, outliers, and inconsistencies. Specifically: (1) For missing data on industrial added value and average number of employees, we used the number of employees at year-end to complete the average number of employees. For missing industrial added value, we used the formula: Industrial Added Value = Total Industrial Output—Intermediate Inputs + VAT Payable. (2) We removed duplicate samples within the same year. (3) We deleted samples where key indicators such as industrial added value or number of employees were missing. (4) We excluded enterprises with fewer than eight employees.

For descriptive statistics results, refer to Table 2.

**Table 2:** Descriptive statistics of main variables.

Type	Symbol	Meaning	Number	Mean	Std. Dev	Min	Max
Explained variable	*TFP*	Total Factor Productivity	782329	5.171	1.217	-6.383	11.630
Control variable	*lnROA*	Return on Assets	764084	0.060	0.149	-7.948	5.678
	*lnmwage*	Wage Cost	764084	7.460	1.426	0.000	16.298
	*lnmtax*	Tax Burden Level	764084	6.369	2.484	0.000	17.039
	*own*	State Capital Share	764084	0.126	0.319	-6.906	8.753
	*lnage*	Firm Age	764084	2.072	0.945	0.000	7.604

### Empirical results analysis

To ensure the scientific validity of our baseline regression results, we conducted necessary statistical tests before formal estimation. These tests included checks for multicollinearity, variable stationarity, F-test, and Hausman test. The test results show that the average Variance Inflation Factor (VIF) is 1.55, indicating no severe multicollinearity among the variable set; the unit root test for the dependent variable (TFP) rejects the null hypothesis of unit roots in all panels at the 1% significance level; the F-test indicates significant time and individual effects; the Hausman test results suggest that a fixed effects model should be chosen.

Table 3 presents the results of testing H1. Columns 1–3 of Table 2 show the estimation results of the DID without control variables, with firm-level control variables, and with both firm-level and regional-level control variables,. Columns 4–6 sequentially report the estimation results based on the PSM-DID, under the same variable conditions. The estimated coefficients of *Treat* × *Post* in each column are significantly positive.

**Table 3:** Basic regression results.

Variable	TFP	TFP	TFP	TFP	TFP	TFP
	(1)	(2)	(3)	(1)	(2)	(3)
*Treat* × *Post*	0.088***	0.171***	0.171***	0.136***	0.184***	0.124***
	(9.16)	(19.29)	(19.05)	(5.16)	(7.35)	(4.60)
*Post*	0.876***	0.252***	0.372***	0.900***	0.410***	1.047***
	(191.25)	(48.40)	(17.79)	(31.27)	(13.90)	(7.18)
*lnROA*		1.360***	1.352***		1.127***	1.132***
		(152.96)	(152.38)		(30.98)	(31.23)
*lnmwage*		0.256***	0.254***		0.217***	0.218***
		(149.30)	(148.89)		(31.06)	(31.24)
*lnmtax*		0.090***	0.090***		0.095***	0.094***
		(146.23)	(146.85)		(40.28)	(40.12)
*own*		-0.094***	-0.098***		-0.128***	-0.142***
		(-15.40)	(-16.18)		(-5.88)	(-6.53)
*lnage*		0.015***	0.015***		0.028***	0.028***
		(7.63)	(7.39)		(3.37)	(3.37)
*lnN*			0.081***			0.100***
			(18.45)			(5.32)
*lngdp*			-0.065***			-0.479***
			(-3.78)			(-3.88)
*TIA*			1.679***			0.484
			(28.63)			(1.62)
*lnscale*			-0.159***			-0.172***
			(-37.16)			(-11.60)
Firm FE	YES	YES	YES	YES	YES	YES
Time FE	YES	YES	YES	YES	YES	YES
*R* ^2^	0.124	0.256	0.262	0.127	0.222	0.227
Num	782329	764084	764084	98903	98903	98903

Note: 1. Numbers in parentheses are t-statistics; 2. *, **, *** indicate significance at the 10%, 5%, and 1% levels, respectively. 3. Due to perfect collinearity, the treat variable has been omitted. This notation applies to all subsequent regression results reported in tables.

According to the results in column six of Table 3, from a statistical significance perspective, the coefficient for *Treat* × *Post* is positive, with a t-value of 4.60 and a p-value of less than 1%. This supports H1 under conventional levels of statistical significance, suggesting that tax incentive policies have increased total factor productivity in enterprises. In terms of practical significance, the coefficient of 0.124 implies that, after controlling for other factors, enterprises affected by tax incentives (treat = 1) have an average TFP that is 0.124 higher than that of enterprises not affected by such policies (treat = 0).

This uniformity across different model specifications and control variable inclusions solidifies the conclusion that VAT reform has played a constructive role in promoting firm productivity in the area.

### Robustness checks

To further validate the robustness of the results, this study employs the PSM method and conducts robustness checks on the matched samples in the following aspects:

#### Controlling for other policy interferences

To mitigate the interference from concurrent policies, this study further controls for province-year and industry-year fixed effects. Given the implementation of a series of debt relief and government subsidy policies in the Northeast during the same period, controlling for government subsidies and the debt-to-asset ratio is crucial. The estimation results, shown in Table 4, indicate that controlling for province-year fixed effects (column 1), industry-year fixed effects (column 2), and further controlling for debt relief and government subsidies (column 3) consistently demonstrate a significant positive effect of VAT reform on productivity, thereby supporting the core conclusion of this study.

**Table 4:** Excluding policy interference.

Variable	Controlling Other Policy Interferences			Controlling income tax incentive	Omitting 2004
	TFP	TFP	TFP	TFP	TFP
	(1)	(2)	(3)	(4)	(5)
*Treat* × *Post*	0.699***	0.534***	0.535***	0.209***	0.221***
	(9.14)	(7.04)	(7.04)	(23.29)	(21.57)
*Post*	0.312***	0.018	0.015	0.338***	0.440***
	(0.37)	(0.02)	(0.02)	(14.94)	(19.05)
Debt relief			0.005		
			(1.17)		
Government subsidies			0.248***		
			(5.45)		
*lnROA*	1.328***	1.326***	1.325***	1.401***	1.376***
	(149.86)	(151.08)	(149.73)	(144.36)	(136.55)
*nmwage*	0.258***	0.258***	0.257***	0.261***	0.256***
	(151.20)	(152.46)	(152.45)	(138.26)	(137.49)
*lnmtax*	0.090***	0.090***	0.090***	0.088***	0.092***
	(148.49)	(149.55)	(149.38)	(133.39)	(134.72)
*own*	-0.076***	-0.082***	-0.082***	-0.085***	-0.094***
	(-12.58)	(-13.66)	(-13.69)	(-12.29)	(-14.50)
*lnage*	0.022***	0.021***	0.020***	0.016***	0.010***
	(11.21)	(10.51)	(10.44)	(7.09)	(4.64)
*lnN*	0.138***	0.022***	0.022***	0.091***	0.076***
	(28.67)	(3.26)	(3.27)	(19.18)	(15.81)
*lngdp*	-0.121	-0.175	-0.174	-0.078***	-0.132***
	(-0.16)	(-0.23)	(-0.23)	(-4.23)	(-6.83)
*TIA*	3.579***	3.337***	3.336***	1.674***	1.887***
	(6.72)	(6.32)	(6.32)	(25.65)	(27.33)
*lnscale*	-0.197***	0.024***	0.023***	-0.177***	-0.156***
	(-45.17)	(3.58)	(3.57)	(-37.84)	(-33.05)
Firm FE	YES	YES	YES	YES	YES
Time FE	YES	YES	YES	YES	YES
province-time FE	YES	YES	YES	NO	NO
industry-time FE	NO	YES	YES	NO	NO
*R* ^2^	0.274	0.289	0.289	0.257	0.276
Num	764084	764084	764084	643034	645531

#### Excluding the impact of concurrent income tax policies in the Northeast

Since only the central region did not implement income tax incentive policies during the same period, eliminating the central region from the analysis allows for isolating the pure policy impact of VAT reform from the concurrent income tax policies in the Northeast. The estimation results, shown in column 4 of Table 4, where the coefficient for Treat×Post is significantly positive, support the core conclusion of this paper.

#### Omitting the Year 2004

Considering that the VAT reform policy was implemented in the second half of 2004 and the application process for VAT reform was complicated, it is possible that some firms were unable to apply in 2004. By excluding the samples from 2004 and retesting, the estimation results, shown in column 5 of Table 4, also support the conclusion of this study.

#### Parallel trends test

**Fig 5:**
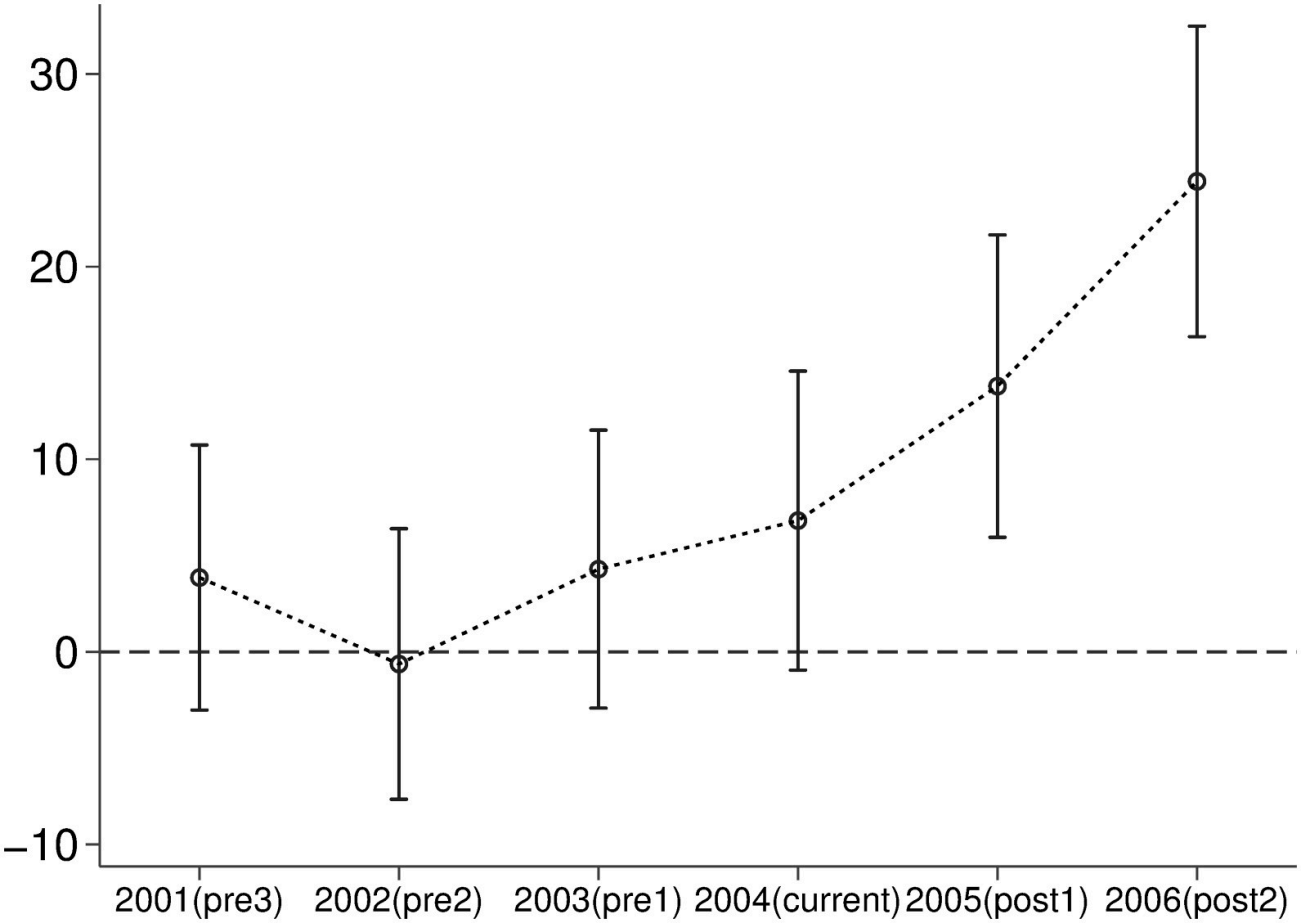
Parallel trends test.

To verify that the VAT reform, as an exogenous shock, had a significantly different impact on the dependent variable before and after its implementation, a parallel trends test is conducted. Specifically, interaction terms are constructed using the dummy variables for the three years before the VAT reform and each year after the reform until the end of the sample period, named as *pre*1, *pre*2, *pre*3, *current*, *post*1, and *post*2 (EndNote 4 in S1 Data). These interaction terms are then included in a fixed effects regression. The results show that the coefficients for *pre*1, *pre*2, and *pre*3 are near zero and not significant, while the coefficients for current, *post*1 and *post*2 are significantly greater than zero (as seen in Fig 5). The parallel trends test, as illustrated in Fig 5), confirms the absence of significant differences in TFP between the treatment and control groups before the VAT reform, with a significant increase in productivity for the treatment group following the policy implementation, thus validating the existence of parallel trends.

### Pacebo test

**Fig 6:**
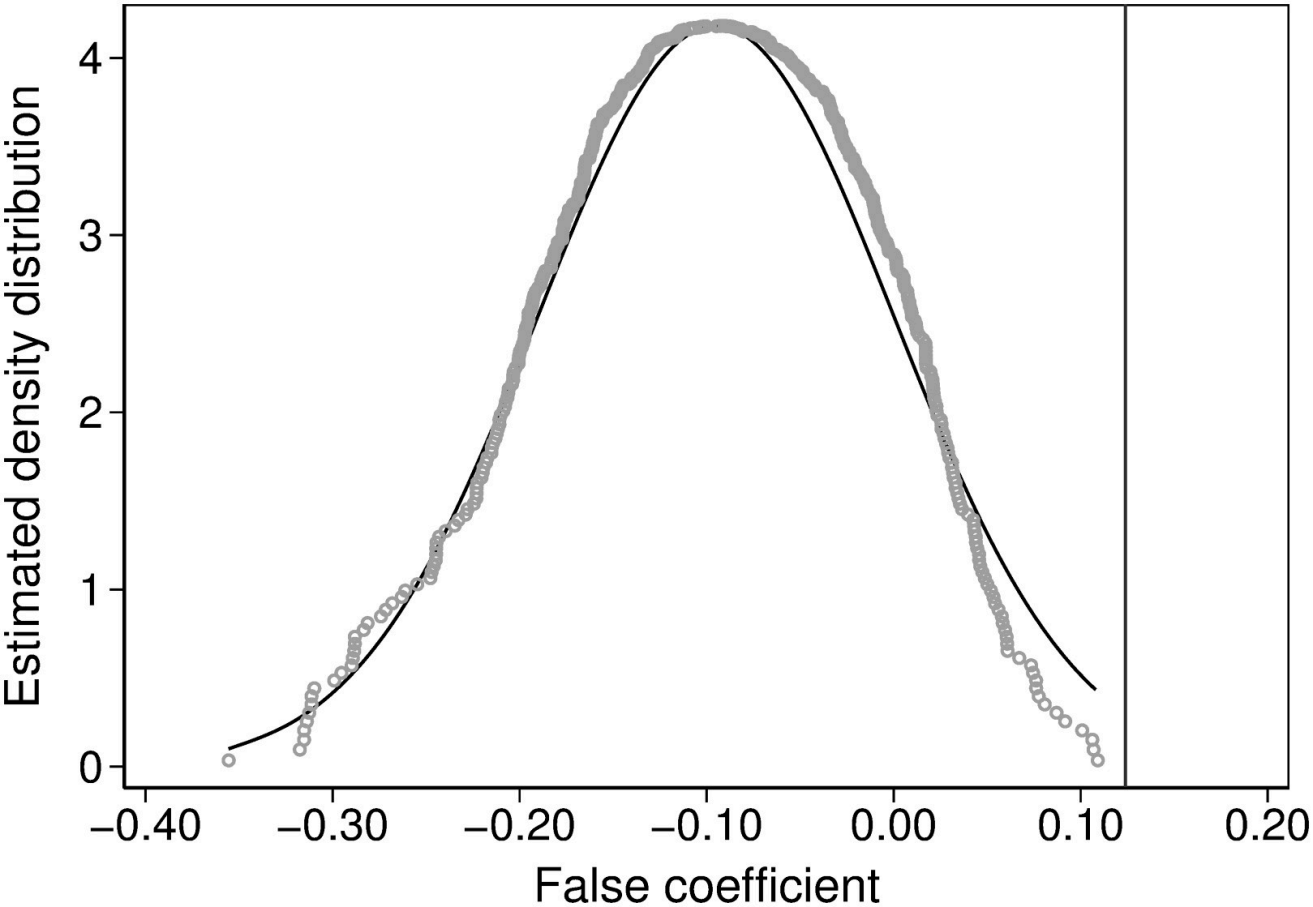
Placebo test.

Following Liu and Sun (2021) [46], we conducted a placebo test by fabricating the policy implementation time and the policy treatment group. We set the policy shock time to the year 2000 and randomly selected the treatment group to test the baseline empirical results of this paper. Our research sample includes eligible enterprises from 31 provincial-level administrative regions in China. Among these, enterprises from three provinces (Liaoning, Jilin and Dalian) form the treatment group. We then randomly selected three provinces from the 31 provincial-level administrative regions as the false treatment group. Finally, we generated the variables *treat*, *post*, and *treat* × *post* based on the fabricated policy shock time and treatment group to conduct the placebo test. We repeated this process 500 times, extracted the false regression coefficients, and plotted the kernel density graph (see Fig 6).

## Mechanism of impact and heterogeneity discussion

### Study on the mechanism of impact

While the robust analysis has proven that regional tax incentives, exemplified by the VAT reform, have enhanced productivity, the intrinsic mechanisms through which tax incentives affect productivity warrant further investigation.

#### Impact of regional tax incentives on investment scale and efficiency

Regional tax incentives have reduced the cost of investment expansion for firms, potentially impacting both the scale and efficiency of their investments. On one hand, this study utilizes fixed capital investment as a proxy for investment scale, with the current investment level of a firm measured by the logarithm of investment (*lninvest*). On the other hand, drawing upon the research by Stein(2003) [47] and Chen et al. (2011) [48], the lagged one-period log value of return on assets (*lnROA*_*t*−1_) is employed to gauge investment opportunities for firms. The following econometric model is constructed to examine the impact of VAT reform on the scale and efficiency of firm investments:

$$\begin{array}{cc} lninvest_{ijkt} & =\varphi_{0}+\varphi_{1}Post+\varphi_{2}Treat+\varphi_{3}lnROA_{ijkt-1}+\varphi_{4}Treat\times Post+\\ & \varphi_{5}Treat\times lnROA_{ijkt-1}+\varphi_{6}Post\times lnROA_{ijkt-1}+\\ & \varphi_{7}Treat\times Post\times lnROA_{ijkt-1}+\Gamma V+\theta_{i}+\gamma_{t}+\varepsilon_{ijkt} \end{array}$$ (3)

In Eq (3), coefficients *φ*_4_ and *φ*_7_ are of particular interest. A positive *φ*_4_ indicates that regional tax incentives, represented by VAT reform, encourage firm investment; a negative *φ*_7_ suggests that these incentives decrease investment efficiency for firms with high investment levels.

Table 5 reports the estimation results based on Eq (3). From a statistical significance perspective, in Table 4, the coefficient for *Treat* × *Post* is positive, with a t-value of 4.29 and a p-value of less than 1%. The coefficient for *Treat* × *Post* × *lnROA*_*t*−1_ is negative, with a t-value of -2.63 and a p-value of less than 1%. This indicates that, at conventional levels of statistical significance, tax incentive policies have increased the scale of corporate investment but have reduced investment efficiency. These empirical results suggest that, on one hand, regional tax incentives under the “Revitalize Northeast China Strategy” have encouraged firms to increase fixed asset investment and technological upgrades, thereby promoting improvements in TFP. On the other hand, regional tax incentives may have reinforced the “soft budget constraints” and “investment hunger syndrome” prevalent among enterprises in Northeast China. Supported by regional tax incentives, enterprises in this region may engage in indiscriminate expansion, thereby reducing investment efficiency.

**Table 5:** Mechanism test for investment scale and efficiency.

Variable	lninvest
	(1)
*Treat* × *Post*	0.182***
	(4.29)
*Post*	-0.513***
	(-6.21)
*Treat* × *Post* × *lnROA*_*t*−1_	-0.822***
	(-2.63)
*Treat* × *lnROA*_*t*−1_	-0.074
	(-0.29)
*Post* × *lnROA*_*t*−1_	0.799***
	(10.74)
*lnROA*_*t*−1_	0.353***
	(5.50)
*lnROA*	-0.018
	(-0.36)
*nmwage*	0.395***
	(44.85)
*lnmtax*	0.037***
	(13.08)
*own*	-0.155***
	(-5.06)
*lnage*	-0.175***
	(-16.47)
*lnN*	0.016
	(0.81)
*lngdp*	0.615***
	(8.03)
*TIA*	1.954***
	(7.15)
*lnscale*	0.038**
	(1.98)
Firm FE	YES
Time FE	YES
*R* ^2^	0.038
Num	327588

#### Are regional tax incentives conducive to innovation?

Innovation is key to sustaining long-term growth in the manufacturing sector, and the prolonged enhancement of TFP is inseparable from a firm’s innovative vitality. On one hand, innovation entails significant risks and uncertainties. On the other hand, corporate innovation relies heavily on investment in technical personnel and experimental materials, demanding high cash flow. Due to challenges like difficulty in measurement and information asymmetry, businesses struggle to use innovation as collateral to secure sufficient external financing [32]. Therefore, innovation relies more on internal corporate funds. Tax incentives have become a crucial policy tool for stimulating innovation. However, in the context of the Northeast region, which bears a significant legacy of the planned economy, can this policy significantly enhance innovation within enterprises of the old industrial base?

This study merges firm data with patent data to empirically examine the impact of regional tax incentives on the logarithmic values of firm applications for invention patents, utility model patents, design patents, and the total number of these three types of patent applications. In Table 6, column 1, the coefficient for *Treat* × *Post* is negative, with a t-value of -0.65 and a p-value greater than 10%. This indicates that at conventional levels of statistical significance, we do not have sufficient evidence to support the hypothesis that tax incentive policies significantly impact the number of total patents in enterprises. Similarly, the empirical results in columns 3 and 4 show that there is insufficient evidence to support the hypothesis that tax incentives significantly affect the number of Utility patents and Design patents respectively. In column 2, the coefficient for *Treat* × *Post* is negative, with a t-value of -2.03 and a p-value of less than 5%, suggesting that tax incentives have reduced the number of Invention patents at a statistically significant level. From a practical significance perspective, the results show that regional tax incentives have not significantly promoted utility model patents, design patents, or the total number of patent applications. There is even a suppressive effect on substantial innovation, such as invention patents. Empirical results indicate that regional tax incentives have a very limited effect on stimulating innovation in the specific area of the old industrial base in Northeast China. Unlike the “rust belt” regions in market economies, such as Detroit in the United States, the old industrial base in Northeast China emerged during the planned economy era, spearheading the prioritized development of heavy industry. This region exhibits strong “path dependence” on the planned economic system. Massive capital investments were a hallmark of economic development in the Northeast’s old industrial base during the planned economy period. Under the “Revitalize the Northeast” policy framework, tax incentives have eased financing constraints for businesses in this region, but companies may be more inclined to make large-scale investments in tangible production equipment rather than engage in higher-risk innovative activities. However, innovation is crucial for the revitalization of old industrial bases. Based on these conclusions, we believe that technology embedded in capital is the primary channel through which regional tax incentives enhance productivity. Therefore, regional tax incentives have failed to place the Northeast region on a path of endogenous growth.

**Table 6:** Mechanism test for corporate innovation.

Variable	Total patents	Invention patents	Utility patents	Design patents
	(1)	(2)	(3)	(4)
*Treat* × *Post*	-0.032	-0.051**	-0.062	0.023
	(-0.65)	(-2.03)	(-1.60)	(0.68)
*Post*	-0.192*	0.349***	-0.071	-0.190***
	(-1.90)	(6.78)	(-0.89)	(-2.69)
*lnROA*	0.096	0.005	0.085*	-0.000
	(1.59)	(0.16)	(1.76)	(-0.01)
*nmwage*	0.106***	0.050***	0.067***	0.041***
	(10.46)	(9.69)	(8.29)	(5.86)
*lnmtax*	0.009***	0.005***	0.007**	0.002
	(2.89)	(3.18)	(2.52)	(0.80)
*own*	-0.115***	-0.020	-0.045**	-0.043**
	(-4.02)	(-1.35)	(-1.99)	(-2.17)
*lnage*	-0.135***	-0.035***	-0.061***	-0.073***
	(-10.39)	(-5.33)	(-5.95)	(-8.03)
*lnN*	0.001	-0.002	0.004	-0.000
	(0.14)	(-0.60)	(0.67)	(-0.07)
*lngdp*	-0.199**	-0.167***	-0.111*	-0.045
	(-2.40)	(-3.95)	(-1.69)	(-0.77)
*TIA*	0.362	-0.190	0.368	0.401*
	(1.22)	(-1.26)	(1.56)	(1.94)
*lnscale*	0.010	0.003	0.006	0.006
	(1.24)	(0.69)	(0.98)	(0.97)
Firm FE	YES	YES	YES	YES
Time FE	YES	YES	YES	YES
*R* ^2^	0.018	0.031	0.007	0.012
Num	42863	42863	42863	42863

Tables 5 and 6 present the results for testing H2. The results indicate that regional tax incentives have alleviated financing constraints for enterprises in the old industrial base, further stimulating their investment motivation. However, with the support of tax incentives, enterprises are more inclined to pursue expansion of scale rather than enhancing riskier inventive innovation. Based on these conclusions, we believe that investment embedded with technological progress is the primary mechanism through which regional tax incentives enhance productivity. The regional tax incentives have not significantly fostered investment efficiency or innovation, and may even have a suppressive effect.

### Heterogeneity analysis of the impact of regional tax incentives on productivity

Table 7 provides the test results for H3.

**Table 7:** Heterogeneity analysis.

	Financing Constraints		Ownership Types		Market Power	
Variable	High constraint	Low constraint	SOEs	Non-SOEs	Monopolistic	Non-monopolistic
	TFP	TFP	TFP	TFP	TFP	TFP
	(1)	(2)	(3)	(4)	(5)	(6)
*Treat* × *Post*	0.163***	0.137***	0.215***	0.153***	0.183***	0.155***
	(13.00)	(8.91)	(7.21)	(15.99)	(12.87)	(12.14)
*Post*	0.417***	0.499***	0.292***	0.411***	0.553***	0.326***
	(14.65)	(13.71)	(3.78)	(18.68)	(16.60)	(11.03)
*lnROA*	1.312***	1.267***	0.965***	1.408***	1.245***	1.333***
	(96.68)	(100.29)	(30.66)	(153.20)	(99.06)	(93.12)
*nmwage*	0.232***	0.230***	0.288***	0.246***	0.214***	0.283***
	(91.84)	(87.71)	(49.89)	(137.16)	(87.40)	(103.35)
*lnmtax*	0.098***	0.074***	0.118***	0.084***	0.078***	0.093***
	(101.65)	(86.85)	(57.76)	(131.43)	(87.45)	(99.05)
*own*	-0.094***	-0.036***	-0.139***	-0.080***	-0.067***	-0.093***
	(-12.73)	(-3.02)	(-3.28)	(-3.00)	(-5.99)	(-12.01)
*lnage*	0.019***	0.111***	0.010	0.026***	0.025***	0.003
	(4.65)	(23.00)	(1.54)	(12.14)	(8.41)	(1.01)
*lnN*	0.047***	0.145***	0.110***	0.078***	0.094***	0.064***
	(7.96)	(18.77)	(7.74)	(16.70)	(14.19)	(9.50)
*lngdp*	-0.089***	-0.064**	-0.084	-0.071***	-0.078***	-0.075***
	(-3.78)	(-2.23)	(-1.30)	(-3.99)	(-2.88)	(-3.06)
*TIA*	1.604***	1.691***	1.256***	1.729***	1.702***	1.314***
	(19.92)	(17.26)	(6.18)	(27.92)	(18.53)	(15.66)
*lnscale*	-0.160***	-0.154***	-0.095***	-0.173***	-0.160***	-0.147***
	(-27.07)	(-21.69)	(-6.55)	(-38.39)	(-24.80)	(-22.88)
Firm FE	YES	YES	YES	YES	YES	YES
Time FE	YES	YES	YES	YES	YES	YES
*R* ^2^	0.223	0.293	0.161	0.279	0.276	0.241
Num	383350	380734	97541	666543	383329	380755

#### Discussion based on different financing constraints

Financing constraints significantly restrict companies, particularly those in manufacturing, from undertaking technological upgrades and transformations. Post the 1990s, firms in the Northeast faced operational difficulties, with financing constraints being a critical barrier to their development. Represented by VAT reform, regional tax incentives aimed to lessen the cost burden of corporate investments, anticipated to notably benefit firms under higher financing constraints. Drawing on Hadlock and Pierce(2010) [49] to construct the SA index, a measure of a firm’s financing constraints level, firms above the median are classified as having high financing constraints, whereas those below as having low.

In Table 7, statistically, for enterprises with high financing constraints, the Treat × Post coefficient is 0.163 (t-value of 13.00, significant at the 1% level); for enterprises with low financing constraints, the Treat × Post coefficient is 0.137 (t-value of 8.91, significant at the 1% level). Practically speaking, the tax incentive policy has a greater promotional effect on the TFP of enterprises with high financing constraints than on those with low financing constraints. Enterprises with higher financing constraints face greater difficulties in promoting technological upgrades before receiving tax incentives. Under the regional tax incentives, the investment costs for these enterprises decrease, aiding them in enhancing technological transformations and improving their TFP.

#### Discussion based on different ownership types

Unlike “Rust Belts” in many parts of the world, which emerged during the industrial revolutions, China’s old industrial base rose during the planned economy era, with state-owned enterprises (SOEs) being its backbone. In Table 7, statistically, for state-owned enterprises, the *Treat* × *Post* coefficient is 0.215 (t-value of 7.21, significant at the 1% level); for non-state-owned enterprises, the *Treat* × *Post* coefficient is 0.153 (t-value of 15.99, significant at the 1% level). Practically speaking, the tax incentive policy has a greater effect on the TFP of state-owned enterprises than on non-state-owned enterprises. In the Northeast region, with its strong legacy of a planned economy, many state-owned enterprises are subject to ‘soft budget constraints,’ and the tax incentives further enhance their investment behavior. This leads to large-scale purchases of advanced equipment, effectively improving these enterprises’ short-term production efficiency and thereby increasing their TFP.

#### Discussion based on firm market power

Many old industrial bases globally developed during the first and second industrial revolutions, with scale expansion and cost reduction as their development strategy. Such areas often house a number of monopolistic firms, leading to a lack of competition in the product market. For instance, Alder et al. (2014) [50] highlighted how powerful labor unions and oligopolistic firms distorted market competition, contributing to the stagnation of “Rust Belts”. This issue also persists in the Northeast’s old industrial base. This section focuses on the relationship between regional tax incentives and TFP across firms with different market standings. Following Peress(2010) [51], the ratio of “(revenue—cost of goods sold—selling expenses—administrative expenses) / revenue” serves as a proxy for firm market power. Firms with this ratio above the median are considered monopolistic.

In Table 7, statistically, for monopolistic enterprises, the *Treat* × *Post* coefficient is 0.183 (t-value of 12.87, significant at the 1% level); for non-monopolistic enterprises, the *Treat* × *Post* coefficient is 0.155 (t-value of 12.14, significant at the 1% level). Practically speaking, the tax incentive policy has a greater effect on the TFP of monopolistic enterprises than on non-monopolistic enterprises. Monopolistic enterprises often have considerable strength. Under the tax incentives of the VAT reform in the Northeast, these enterprises are more motivated to expand their scale and invest in advanced equipment. As a result, the tax incentives have a more significant impact on the TFP of monopolistic enterprises.

## Regional tax incentives, enterprise dynamics evolution, and manufacturing productivity

### Impact of regional tax incentives on manufacturing sector productivity through the lens of firm dynamics

The empirical results discussed thus far analyze the impact of VAT reforms, representing regional tax incentives, on the TFP of firms in the Northeast region from a microeconomic perspective. This paper aims to further investigate the effect of regional tax incentives on the industry-level productivity in the manufacturing sector from the perspective of firm dynamics.

### DOP decomposition of TFP in the old industrial bases of the Northeast

Building on the static decomposition framework by Olley and Pakes(1996) [52], Melitz and Polanec(2015) [26] introduced intertemporal entry and exit of firms, proposing the DOP decomposition model. Utilizing the DOP model, the dynamic components of productivity changes can be delineated as follows:

$$\begin{array}{ll} \Delta \Phi & =\left(\Phi_{S2}-\Phi_{S_{1}}\right)+s_{E_{2}}\left(\Phi_{E_{2}}-\Phi_{S_{2}}\right)+s_{X1}\left(\Phi_{S_{1}}-\Phi_{X_{1}}\right)↓\\ & =\underset{\begin{array}{c} \text{Within-Firm}\\ \text{Growth} \end{array}}{\underset{︸}{\Delta {\bar{\varphi }}_{S}}}+\underset{\text{Reallocations}}{\underset{︸}{\underset{\begin{array}{c} \text{Between-Firm}\\ \text{Reallocation} \end{array}}{\underset{︸}{\Delta \;{\text{cov}}_{S}}}+\underset{\text{Entry}}{\underset{︸}{s_{E_{2}}\left(\Phi_{E_{2}}-\Phi_{S_{2}}\right)}}+\underset{\text{Exit}}{\underset{︸}{s_{X_{1}}\left(\Phi_{S_{1}}-\Phi_{X_{1}}\right)}}}} \end{array}$$ (4)

In Eq (4), $\Phi_{t}=\sum_{i}\;s_{it}\varphi_{it}={\bar{\varphi }}_{t}+\sum \left(s_{it}-{\bar{s}}_{t}\right)\left(\varphi_{it}-{\bar{\varphi }}_{t}\right)={\bar{\varphi }}_{t}+cov\left(s_{it},\varphi_{it}\right)$ represents the aggregate productivity. Here, *φ*_*it*_ denotes the productivity of firm *i*, ${\bar{\varphi }}_{t}=\frac{1}{n_{t}}\sum_{i=1}^{n_{t}}\varphi_{it}$ is the simple average productivity, ${\bar{s}}_{t}=\frac{1}{n_{t}}\sum_{i=1}^{n_{t}}s_{it}=\frac{1}{n_{t}}$ is the simple average market share, and ΔΦ signifies the difference in aggregate productivity between two periods. $\Phi_{\text{Gt}}=\sum_{i\in G}(\frac{s_{it}}{s_{Gt}})\varphi_{it}$ indicates the weighted average productivity level of different categories of firms, where *s*_*Gt*_ = ∑_*i*∈*G*_ *s*_*it*_ is the total market share of each firm category, denoted by G, representing surviving (S), entering (E), and exiting (X) firms. The categorization of firms into entering and exiting statuses is based on the prior year in this paper. Following the approach of Melitz and Polanec(2015) [26], the aggregate productivity growth of regional manufacturing is decomposed from the perspective of firm productivity dynamics into four sources: the contribution of productivity improvement within surviving firms to aggregate productivity (Within-Firm Growth), the contribution of resource allocation efficiency improvement between surviving firms to aggregate productivity (Between-Firm Reallocation), the contribution of high-efficiency firm entry to aggregate productivity (Entry), and the contribution of low-efficiency firm exit to aggregate productivity (Exit). The sum of the contributions from Between-Firm Reallocation, high-efficiency firm entry, and low-efficiency firm exit constitutes the improvement in aggregate resource allocation efficiency (Reallocations). When Between-Firm Reallocation (Δcov_*s*_) is positive, it indicates that surviving firms with higher productivity have gained greater market share.

### Empirical analysis of regional tax incentives on aggregate manufacturing productivity and its sources

To more accurately examine the impact of VAT reform on the growth and sources of aggregate productivity in the manufacturing sector, the following econometric model was established for estimation:

$$\begin{array}{c} Y_{jkt}=\psi_{0}+\psi_{1}Post+\psi_{2}Treat+\psi_{3}Treat\times Post+\Psi Z+\theta_{jk}+\gamma_{t}+\varepsilon_{jkt} \end{array}$$ (5)

In Eq (5) Y represents the components of growth in aggregate productivity in the manufacturing sector, and Z denotes province-level control variables. The meanings of other variables are consistent with those in previously described estimation models and are not reiterated here.

Table 8 provides the test results for H4 and H5. Specifically, it examines the impact of tax incentives on the growth and sources of Aggregate Productivity across six major industries. For Aggregate Productivity, the *Treat* × *Post* coefficient is 0.106 (t-value of 2.64, significant at the 1% level); for Within-Firm Growth, the *Treat* × *Post* coefficient is also 0.064 (t-value of 2.43, significant at the 5% level); for Reallocations, the *Treat* × *Post* coefficient is -0.009 (t-value of -0.21, not significant); for Between-Firm Reallocations, the *Treat* × *Post* coefficient is 0.042 (t-value of 1.24, not significant); for Entry, the *Treat* × *Post* coefficient is -0.018 (t-value of -1.78, significant at the 10% level); and for Exit, the *Treat* × *Post* coefficient is -0.033 (t-value of -1.82, significant at the 10% level). Practically speaking, the tax incentive policy has not significantly improved the efficiency of resource allocation and has suppressed the effects of low-efficiency enterprise exits and high-efficiency enterprise entries on Aggregate Productivity growth. Regional tax incentives have limited the market mechanism’s role in natural selection, hindering optimal resource allocation. Additionally, regional tax incentives have limited appeal to high-efficiency enterprises and have failed to effectively create an agglomeration economy. Although in the short term, regional tax incentives have promoted an increase in TFP at the enterprise level, the distortion in resource allocation is ultimately detrimental to the endogenous sustainable growth of the Northeast region’s economy in the long term.

**Table 8:** Estimation of regional tax incentives on dynamic changes in aggregate productivity.

Variable	Aggregate Productivity	Within-Firm Growth	Reallocations	Between-Firm Reallocations	Entry	Exit
	(1)	(2)	(3)	(4)	(5)	(6)
*Treat* × *Post*	0.106***	0.064**	-0.009	0.042	-0.018*	-0.033*
	(2.64)	(2.43)	(-0.21)	(1.24)	(-1.78)	(-1.82)
*Post*	0.162	0.292***	-0.174**	-0.130*	-0.045	0.000
	(1.53)	(3.78)	(-2.02)	(-1.68)	(-1.06)	(0.01)
*lnN*	-0.017	-0.040	-0.002	0.023	-0.002	-0.022
	(-0.42)	(-1.21)	(-0.09)	(0.96)	(-0.33)	(-1.60)
*lngdp*	-0.120	-0.126*	-0.039	0.006	0.004	-0.049
	(-1.21)	(-1.81)	(-0.45)	(0.08)	(0.12)	(-1.01)
*TIA*	0.809**	0.763***	0.085	0.046	-0.048	0.088
	(2.57)	(2.94)	(0.27)	(0.18)	(-0.50)	(0.51)
*lnscale*	0.123***	0.057**	0.126***	0.065**	0.023**	0.038***
	(3.55)	(2.10)	(4.20)	(2.48)	(1.99)	(2.75)
Firm FE	YES	YES	YES	YES	YES	YES
Time FE	YES	YES	YES	YES	YES	YES
*R* ^2^	0.112	0.199	0.032	0.011	0.063	0.026
Num	2125	2125	2125	2125	2125	2125

### Re-discussion of the resource allocation effect of regional tax incentives: A focus on “zombie firms”

Our further investigation delves into the creation probability of “zombie firms” within the Northeast region, examining how regional tax incentives might distort resource allocation. “zombie firms” not only lack self-sufficiency but also hoard substantial production factors, detrimentally affecting regional productivity. This paper studies the impact of VAT reform on the probability of “zombie firms” emergence, establishing the following model:

$$\begin{array}{c} zombie_{ijkt}=\omega_{0}+\omega_{1}Post+\omega_{2}Treat+\omega_{3}Treat\times Post+\Omega V+\theta_{i}+\gamma_{t}+\varepsilon_{ijkt} \end{array}$$ (6)

In Eq (6), *zombie* is a dummy variable, assigned a value of 1 for “zombie firm”, otherwise 0. Drawing on the identification methods of Caballero et al. (2008) [53] and Fukuda and Nakamura (2011) [54], a firm is considered a “zombie firm” if its total annual profit falls below net interest expenses, total debt increases compared to the previous year, and the asset-liability ratio exceeds 0.5.

Table 9 provides further evidence supporting hypothesis H5. Statistically, the coefficient of *Treat* × *Post* is positive, with a t-value of 3.07 and a p-value of less than 1%. This indicates that, at standard levels of statistical significance, the tax incentive policy has increased the likelihood of zombie company formation. In practical terms, regional tax incentives have continuously injected resources into inefficient enterprises, stimulating investment behavior in businesses that are already struggling, thereby increasing their operational risks. As a result, this policy has heightened the probability of the emergence of zombie companies in Northeast China. These findings further support the notion that regional tax incentives have hindered the exit of inefficient enterprises in the region. Since zombie companies occupy local economic resources, they further deteriorate the local business environment, adversely affecting the attraction of efficient enterprises.

**Table 9:** Regional tax incentives & “zombie firms”.

Variable	zombine
	(1)
*Treat* × *Post*	0.013***
	(3.07)
*Post*	0.339***
	(34.82)
*lnROA*	-0.442***
	(-106.67)
*nmwage*	-0.007***
	(-8.21)
*lnmtax*	-0.008***
	(-28.65)
*own*	0.032***
	(11.20)
*lnage*	0.023***
	(24.54)
*lnN*	-0.015***
	(-7.14)
*lngdp*	-0.023***
	(-2.83)
*TIA*	0.006
	(0.22)
*lnscale*	-0.005**
	(-2.34)
Firm FE	YES
Time FE	YES
*R* ^2^	0.076
Num	764084

### Impact of regional tax incentives on resource allocation in capital and technology-intensive industries

The Northeast industrial base, a cornerstone in China’s industrialization journey, has historically developed into three main categories of industries. The first category comprises resource-dependent industries, which, due to the depletion of local resources, are now facing a phase of managed decline. The second category includes industries that rely on both capital and resources, such as Ansteel, Jilin Ferroalloy, Jilin Carbon, and Fushun Aluminum Factory, which are engaged in fierce market competition. The third category consists of industries primarily dependent on technology and capital, notably equipment manufacturing, shipbuilding, and the automotive industry. These industries not only have high barriers to entry in terms of capital and technology, making many of their products critical components of national power, but they also represent a vital foundation for building a high-level industrial system in China. Furthermore, the Northeast industrial base benefits from a solid foundation in related industries and human capital. The revitalization of these industries is crucial for the rejuvenation of the old industrial base. Whether regional tax incentives can optimize resource allocation in these industries and enhance aggregate productivity remains an area for further research.

Table 10 presents further validation results for H4 and H5. For Reallocations, the coefficient of *Treat* × *Post* is -0.09 (with a t-value of -2.08, significant at the 5% level); for Exit, the *Treat* × *Post* coefficient is -0.07 (with a t-value of -3.05, significant at the 1% level). This indicates that regional tax incentives also exert a restraining effect on resource allocation in these industries, particularly in significantly inhibiting the exit of inefficient enterprises. These enterprises typically demand high levels of capital and technology, and regional tax incentives, represented by value-added tax reforms, provide stronger investment incentives for them. However, such preferential policies also intensify the transfusion of resources to inefficient enterprises, alleviating their survival pressure, ultimately leading to limited effects on aggregate productivity enhancement.

**Table 10:** Estimation of regional tax incentives on dynamic changes in aggregate productivity for capital and technology-intensive industries.

Variable	Aggregate Productivity	Within-Firm Growth	Reallocations	Between-Firm Reallocations	Entry	Exit
	(1)	(2)	(3)	(4)	(5)	(6)
*Treat* × *Post*	0.040	0.043	-0.090**	-0.003	-0.016	-0.070***
	(0.91)	(1.08)	(-2.08)	(-0.09)	(-1.17)	(-3.05)
*Post*	0.185	0.316***	-0.096	-0.131	-0.076	0.111*
	(1.05)	(2.72)	(-1.04)	(-1.18)	(-1.04)	(1.74)
*lnN*	-0.035	-0.052	0.016	0.017	0.009	-0.010
	(-0.86)	(-1.52)	(0.66)	(0.63)	(1.11)	(-0.69)
*lngdp*	-0.135	-0.127	-0.110	-0.008	0.026	-0.128**
	(-0.83)	(-1.21)	(-1.12)	(-0.08)	(0.38)	(-2.39)
*TIA*	0.305	0.360	0.157	-0.055	-0.031	0.242
	(0.77)	(1.17)	(0.42)	(-0.18)	(-0.23)	(0.96)
*lnscale*	0.144***	0.044**	0.150***	0.100***	0.027*	0.023
	(4.19)	(2.06)	(4.62)	(3.48)	(1.71)	(1.42)
Firm FE	YES	YES	YES	YES	YES	YES
Time FE	YES	YES	YES	YES	YES	YES
*R* ^2^	0.141	0.212	0.057	0.032	0.049	0.022
Num	1088	1088	1088	1088	1088	1088

## Conclusion and discussion

### Results

The Value-Added Tax (VAT) reform initiated in the Northeast region is a crucial measure within the “Revitalize the Northeast” strategy. By transitioning from a “production-based VAT” to a “consumption-based VAT”, manufacturing and related industries in the Northeast region have enjoyed the benefits of tax incentives. Through empirical research, we have discovered the following results: (1) Regional tax incentives significantly increased enterprise productivity. (2) Compared to the control group, the experimental group showed a decrease in sensitivity to investment opportunities. Furthermore, regional tax incentives did not significantly increase patent output among enterprises. (3) Regional tax incentives have alleviated financing constraints for enterprises in old industrial bases, significantly enhancing the TFP of enterprises with higher financing constraints. Additionally, the policy has had a stronger promotional effect on the TFP of state-owned and monopolistic enterprises. (4) From the perspective of enterprise dynamic evolution, tax incentive policies impeded the improvement of regional resource allocation efficiency. (5) Regional tax incentive policies increased the likelihood of creating “zombie firms”.

### Research contributions

The study enriches the literature on the effects of tax incentives on the productivity of enterprises in old industrial bases. Previous research on the exogenous shocks impacting enterprise productivity rarely includes the regional background of enterprises [43, 45] and few studies have deeply discussed the differential impacts of regional tax incentives on input-embedded TFP and input-free TFP [30, 55]. Regional tax incentives are crucial policy tools for achieving coordinated regional development in developing countries, but their effectiveness in old industrial bases remains a question. This paper systematically investigates this issue by incorporating the unique characteristics of old industrial bases.

The study incorporates the refined dynamic decomposition of productivity by Melitz and Polanec (2015) [26] into the economic performance assessment of regional tax incentives. This approach deepens our understanding of the impact of regional tax incentives on the economic performance of old industrial bases. The DOP method proposed by Melitz and Polanec (2015) [26] is advantageous as it allows for the decomposition of productivity differences into various sources, providing deeper insights. Additionally, research on zombie enterprises enriches the literature related to resource allocation theory. Zombie enterprises are defined as businesses that have ceased production or are partially operational, suffer from consecutive losses, and are insolvent, primarily surviving on government subsidies and bank renewals [56]. Existing studies suggest that the presence of zombie enterprises reduces corporate investment and is detrimental to economic development [57–59]. There is scant literature discussing this from the perspective of resource allocation.

The research further enriches the study of the impacts of regional tax incentives on the productivity of specific types of enterprises and industries. On the one hand, we delve into the impact of regional tax incentives on state-owned and monopolistic enterprises; on the other hand, we analyze the effect of regional tax incentives on resource allocation in capital and technology-intensive industries. State-owned enterprises in China are typically owned or controlled by the government or government-controlled entities. The government can use state-owned enterprises to implement industrial policies, promote the development of strategic industries, and provide employment and stabilize the market during economic downturns. [60–62]. Capital and technology intensive industries are the backbone industries of old industrial areas. Existing studies often focus on the innovation, profitability, and financing capabilities of state-owned enterprises, rarely linking them with the economic conditions of the industry [35, 62].

### Policy recommendation

For old industrial areas, regional tax incentives should be coordinated with market-oriented reforms. Research findings indicate that while regional tax incentives have stimulated corporate investment and enhanced production efficiency, they have also resulted in decreased investment efficiency and a lack of innovation. Unlike regions with well-developed market economies, the old industrial bases in Northeast China still exhibit strong path dependence on the planned economic system. Therefore, regional tax incentives, if not advanced in conjunction with market-oriented reforms, may face greater challenges in achieving expected outcomes over the long term. In the future, to polish the “rust belt”, efforts should focus on promoting market-oriented reforms and leveraging regional tax incentives on the foundation of marketization. Continuing market reforms and creating a top-tier business environment are essential. With a better market-oriented environment, regional tax incentives can better attract highly efficient enterprises, thus generating new momentum to drive the “rust belt” toward rejuvenation.

Regional tax incentives policies should also be enriched to stimulate corporate innovation. Empirical evidence in this article reveals that regional tax incentives have not effectively encouraged corporate innovation, especially in terms of inventive innovation. This is a significant factor contributing to the limited long-term economic policy effects of regional incentives in the Northeast region. In the future, efforts should be focused on continuously deepening market-oriented reforms while further enriching the content of regional tax incentives. Specifically, there should be a strengthening of tax incentives in research and development (R&D) in the old industrial bases of the Northeast to encourage companies to allocate more resources to R&D activities, thereby sparking new momentum for long-term growth in the region. Simultaneously, attention should be given to complementing tax incentives with other policy tools. Additionally, efforts should be directed towards breaking the innovation inertia within Northeastern companies. This involves nurturing entrepreneurial spirit within Northeastern companies, shifting away from a tendency of “emphasizing investment, neglecting innovation”. Differentiated funding policies should also be implemented to encourage innovative initiatives among high-innovation companies in the Northeast, urging those lacking innovation drive to engage in substantive innovative activities. The goal is to create a better environment for regional tax incentives to promote corporate innovation and to facilitate better development in old industrial bases.

Deepen ownership reform to enhance the endogenous capacity of enterprise development. The empirical results in this article demonstrate that under the incentive of regional tax preferences, the status of state-owned enterprises is further strengthened. Compared to private enterprises, state-owned enterprises lack innovation drive. This, to some extent, has become a significant factor constraining the weak endogenous development in the Northeast region. In the future, on one hand, efforts should continuously be made to build a socialized service system supporting non-publicly-owned economies in old industrial bases through entrepreneurship support, technology platforms, investment and financing, etc. This will actively promote the healthy development of mixed-ownership economies and create an environment of orderly competition among enterprises in the Northeast. On the other hand, the government should support and improve the governance model and operational mechanisms of state-owned enterprises, firmly establish their position as market entities, avoid strengthening their monopoly position, and preserve regional market vitality.

Capital and technology intensive industries in old industrial bases should follow market principles. The historically formed capital and technology intensive industries are important comparative advantages of old industrial bases and a crucial foundation for China’s high-quality real economy development. However, empirical evidence in this article reveals that regional tax incentives, by providing life support to inefficient enterprises, have distorted market mechanisms and disrupted the resource allocation for revitalizing crucial industries in the Northeast, especially capital and technology intensive industries. In the future, seizing the important historical opportunity of domestic circulation, high-quality development of capital and technology intensive industries should be pursued to advance the industrial upgrading and modernization of the industrial value chain in the Northeast and across the country. However, for these industries, government support measures, including regional tax incentives, should not become tools for life support to inefficient enterprises or zombie companies. For capital and technology intensive industries, the visible hand of the government should play a better role based on market competition, by stimulating the vitality of existing enterprises, attracting efficient enterprises, and promoting the industry’s path towards endogenous development. This approach will aid in rejuvenating the “rust belt”.

### Limitations and prospects

The limitation of this study lies in its singular focus, which restricts the generalizability of the research findings. The enterprise-related data in this study mainly come from the Chinese Firms Data, encompassing both Chinese state-owned enterprises and large and medium-sized manufacturing enterprises with annual revenue of 5 million yuan or more, widely used in industrial enterprise research. However, relying solely on data from a single emerging country (China) to test research hypotheses may limit the universality of the research results. Future research should aim to collect industrial enterprise data from various old industrial zones in different emerging countries to further test the robustness of the conclusions drawn in this study.

Furthermore, the scope and content of the research can be expanded. This study, from the perspective of resource allocation, demonstrates the impact of tax incentive policies on productivity. However, enhancing regional productivity and ensuring sustainable economic development entail many other features. Although the development of the digital economy was relatively weak during the sample period of this study, in recent years, the digital economy has been rapidly growing, especially with the deepening integration of the real and digital economies. This offers a new perspective for studying the impact of tax incentives on manufacturing TFP. Future research could explore the role of tax incentive policies based on emerging theories of the digital economy. Additionally, our study only considered VAT reform as one type of tax incentive policy. However, tax incentive policies may vary in implementation across different countries. Future research could expand to include more types of tax incentive policies.

Lastly, there is room for improvement in research methodology. We employed regression analysis, a common method in econometrics, to analyze the causal relationships hypothesized in this study. With the advancement of artificial intelligence and big data, the field of econometrics is exploring the use of machine learning methods for causal analysis. Future research could consider using algorithms such as Random Forest or Gradient Boosting Machine to identify which variables have the strongest predictive power for the dependent variable, and thereby infer possible causal relationships.

## Supporting information

S1 Data (PDF)

S1 Fig Graphical abstract. (TIF)

S1 File Data & program. (ZIP)

S2 File Terms explaining. (PDF)
