# Supplementary material for: Do regional tax incentive policies improve productivity?
Source: PLoS One. 2024 Aug 27;19(8):e0307561. doi: 10.1371/journal.pone.0307561 (PMC11349089; doi:10.1371/journal.pone.0307561)
Supplement: S1 Data — (PDF) [file pone.0307561.s001.pdf]

EndNote

EndNote 1

From 1990 to 2003, the Northeast region's economic output increased by 164.81%, below the national growth rate of 207.67%, based on the regional GDP data adjusted with the GDP deflator index from various editions of the China Statistical Yearbook.

EndNote 2

From 2004 to 2013, the economic growth of Northeast China's three provinces outpaced the national average, increasing by 276.18% compared to the national 255.80%. However, from 2014, growth plummeted, consistently ranking among the lowest nationwide. By 2015, growth rates in Heilongjiang, Jilin, and Liaoning dropped to 5.7%, 6.5%, and 3.0% respectively, well below the national average of 6.9%, with Liaoning recording the lowest in China.

EndNote 3

Six major industries: equipment manufacturing (34, 35, 36, 39, 40, 41), petrochemicals (25, 26), metallurgy (32, 33), shipbuilding and auto manufacturing (37), and agricultural processing (13). Military/high-tech production excluded due to definition challenges. Codes follow GB/T4754-2017 classification.

EndNote 4

Parallel trends test formula: $TFP_{\mathrm{ij}kt}^{PSM} = \delta_0 + \delta_1 pre_1 + \delta_2 pre_2 + \delta_3 pre_3 + \delta_4 current + \delta_5 post_1 + \delta_6 post_2 + \Delta\mathrm{X} + \theta_i + \gamma_t + \varepsilon_{it}$. Coefficients for 2001-2006 correspond to $\delta_i, i =, 2, 3, 4, 5, 6$.
