# Supplementary figures and images for: Do regional tax incentive policies improve productivity?
Source: PLoS One. 2024 Aug 27;19(8):e0307561. doi: 10.1371/journal.pone.0307561 (PMC11349089; doi:10.1371/journal.pone.0307561)

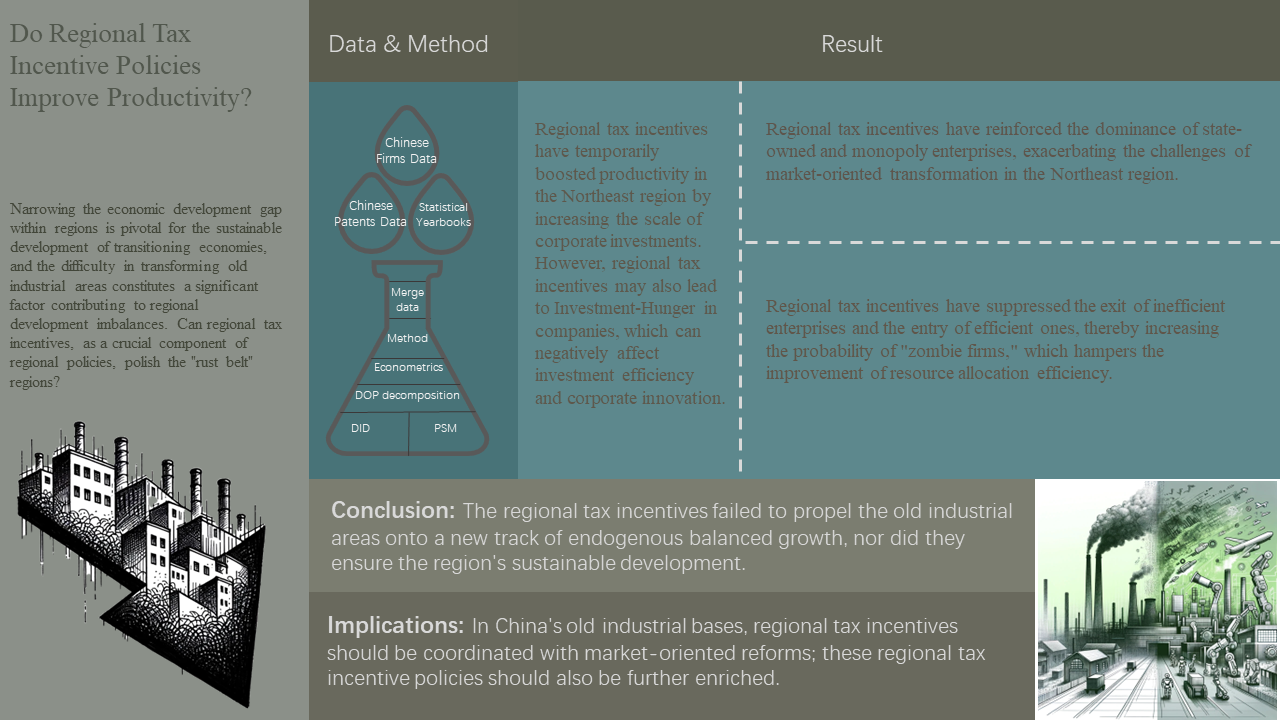

Supplement: S1 Fig — (TIF) [file pone.0307561.s002.tif]
