# Supplementary material for: Do regional tax incentive policies improve productivity?
Source: PLoS One. 2024 Aug 27;19(8):e0307561. doi: 10.1371/journal.pone.0307561 (PMC11349089; doi:10.1371/journal.pone.0307561)
Supplement: S2 File — (PDF) [file pone.0307561.s004.pdf]

Defining Terms

1. Total Factor Productivity (TFP)

Note: Total Factor Productivity (TFP) is often interpreted as the portion of total output that cannot be attributed solely to the input of factors. There are two primary methods for calculating TFP in firms: parametric and non-parametric approaches. Common parametric methods include the OP[1] and LP[2] methods, while popular non-parametric methods are Data Envelopment Analysis (DEA) and Stochastic Frontier Analysis (SFA). Given the focus of this paper on analyzing the relationship between resource allocation and productivity, the non-parametric methods of DEA and SFA are not suitable for our research objectives. Additionally, the OP method, which uses a firm's investment as a proxy variable, helps mitigate issues like simultaneity bias and selectivity and attrition bias inherent in the OLS method. However, there is no uniform method for estimating firm investment. For instance, using the conventional perpetual inventory method can lead to a significant loss of data points and induce selection bias, which is also detrimental to the resource allocation analysis planned for later in the paper. Therefore, this study employs the LP method to estimate TFP.

2. Zombie Firms

Note: Zombie firms are defined as businesses that have ceased or partially ceased operations, consistently incur losses year after year, and have liabilities exceeding assets. They primarily rely on government subsidies and bank loan renewals to sustain their existence and operations [3].

3. Soft Budget Constraints

Note: In socialist economies, when state-owned enterprises incur losses, it is common for the government to respond by increasing investments, extending more loans, reducing taxes, and offering financial subsidies. This phenomenon, referred to by Kornai (1986)[4] as 'soft budget constraints', highlights a lenient fiscal approach towards public enterprises facing financial difficulties.

4. Investment Hunger

Note: Kornai (1986) highlights that in socialist economies, social planners have a strong impulse to invest, vividly described as "investment hunger"[4]. Due to soft budget constraints, enterprises recognize that no matter how costly or extensive their investment mistakes are, these errors will not lead to bankruptcy or liquidation. Additionally, in socialist systems, there are bureaucrats instead of true owners. These bureaucrats are responsible for making investment decisions, yet they do not bear any personal financial consequences for losses incurred. As a result, in the classic socialist system, the demand for investment by enterprises is insatiable.

5. Dynamic Olley-Pakes Decomposition (DOP decomposition)

Note: Aggregate productivity is a weighted average of productivity at the producer level (firm or plant). The enhancement of aggregate productivity results from a combination of firm-level productivity growth, the reallocation of resources between enterprises, and the entry of high-efficiency firms coupled with the exit of low-efficiency ones (Foster et al., 2001; Bartelsman et al.,

2013). Melitz and Polanec (2015)[5] proposed an extension of the productivity decomposition method developed by Olley and Pakes (1996)[1]. This extension provides an accounting for the contributions of entry and exit to aggregate productivity changes; and it also breaks down the separate contributions of firm-level productivity shifts and market share reallocations among surviving firms.

6. Institutional Inertia

Note: It refers to the tendency of institutions, like organizations, government bodies, or companies, to resist changes and continue with their established procedures and practices, even in the face of new challenges or opportunities that call for different approaches.

7. Revitalize the Northeast

Note: The Northeast region is an old industrial area in China, comprising the three provinces of Liaoning, Jilin, and Heilongjiang

8.Production-based VAT and Consumption-based VAT

Note: A "production-based value-added tax" allows for the deduction of taxes included in purchased raw materials but not those included in purchased fixed assets. Conversely, a "consumption-based value-added tax" permits the deduction of taxes on all purchased items, including both raw materials and fixed assets.

9 Five-Year Plan

The Five-Year Plans are a series of social and economic development initiatives issued by the Chinese Communist Party (CCP) since 1953 in the People's Republic of China. Planning is a key characteristic of the nominally socialist economies, and one plan established for the entire country normally contains detailed economic development guidelines for all its regions. In order to more accurately reflect China's transition from a Soviet-style command economy to a socialist market economy (socialism with Chinese characteristics), the plans since the 11th Five-Year Plan for 2006 to 2010 have been referred to in Chinese as "guidelines" instead of as "plans".

References

[1] Olley GS, Pakes A. The Dynamics of Productivity in the Telecommunications Equipment Industry. Econometrica. 1996;64(6):1263–97.

[2] Levinsohn J, Petrin A. Estimating Production Functions Using Inputs to Control for Unobservables. The Review of Economic Studies. 2003;70(2):317–341. doi:10.1111/1467-937X.00246.

[3] Blazkova I, Dvoulety O. Zombies: Who Are They and How Do Firms Become Zombies? JOURNAL OF SMALL BUSINESS MANAGEMENT. 2022;60(1):119–145. doi:10.1080/00472778.2019.1696100.

[4] Kornai J. The Soft Budget Constraint. Kyklos. 1986;39(1):3–30. doi:10.1111/j.1467-6435.1986.tb01252.x.

[5] Melitz MJ, Polanec S. Dynamic Olley-Pakes Productivity Decomposition with Entry and Exit. Rand Jouranl of Economics. 2015;46(2):362–375. doi:10.1111/1756-2171.12088.
